# Transgenic mouse model for the formation of Hirano bodies

**DOI:** 10.1186/1471-2202-12-97

**Published:** 2011-10-06

**Authors:** Sangdeuk Ha, Ruth Furukawa, Michael Stramiello, John J Wagner, Marcus Fechheimer

**Affiliations:** 1 Dept. Cellular Biology, University of Georgia, Athens, GA 30602, USA; 2 Dept. Physiology Pharmacology, University of Georgia, Athens, GA 30602, USA; 3 Beth Israel Deaconess Medical Center, Harvard Medical School, 330 Brookline Avenue, Boston, MA 02215, USA

## Abstract

**Background:**

Hirano bodies are actin-rich cytoplasmic inclusions found predominantly in the brain in association with a variety of conditions including aging and Alzheimer's disease. The function of Hirano bodies in normal aging and in progression of disease has not been extensively investigated due to a lack of experimental model systems. We have developed a transgenic mouse model by expression of a gain-of-function actin cross-linking protein mutant.

**Results:**

We used the Cre/loxP system to permit tissue specific expression of Hirano bodies, and employed the murine Thy 1 promoter to drive expression of Cre recombinase in the brain. Hirano bodies were observed in the cerebral cortex and hippocampus of homozygous double transgenic 6 month old mice containing Cre. The Hirano bodies were eosinophilic rods, and also exhibited the paracrystalline F-actin filament organization that is characteristic of these inclusions. Mice with Hirano bodies appear healthy and fertile, but exhibited some alterations in both short-term and long-term synaptic plasticity, including paired-pulse depression rather than facilitation, and decreased magnitude of early LTP.

**Conclusions:**

Hirano bodies are not lethal and appear to have little or no effect on histology and tissue organization. Hirano bodies do modulate synaptic plasticity and exert clearly discernable effects on LTP and paired-pulse paradigms. This model system will allow us to investigate the impact of Hirano bodies *in vivo*, the pathways for formation and degradation of Hirano bodies, and whether Hirano bodies promote or modulate development of pathology and disease progression.

## Background

Hirano bodies are cytoplasmic, eosinophilic, rod-shaped inclusions that were initially described in patients with amyotrophic lateral sclerosis or Parkinsonism-dementia complex on Guam [1]. Hirano bodies are paracrystalline and are composed of 8-10 nm filaments with 10-12 nm spacing in longitudinal section, or a herringbone pattern in an oblique section [2-4]. Hirano bodies contain F-actin, actin-associated proteins such as tropomyosin, vinculin, and cofilin [5-8], as well as microtubule-associated proteins including tau [9,10]. They also contain a number of other components including a wide variety of growth factors, enzymes, and transcriptional regulators including inducible nitric oxide synthase [11], FAC1 [12], and the cytoplasmic fragment of the amyloid precursor protein [13,14]. These inclusions have been found predominantly in dendrites and cell bodies of hippocampal pyramidal neurons of the brain under various pathological conditions including Alzheimer's disease (AD), Amyotrophic Lateral Sclerosis (ALS), and Parkinson's disease (PD) as well as in normal aged individuals [15,16]. They have also been observed for human and animal pathological material in the cerebral cortex, cerebellum, Purkinje cells, peripheral nerves, oligodendroglia and Schwann cells, astrocytoma, and muscle cells [4]. Further, some model APP transgenic mice accumulate cytoplasmic aggregations structurally similar to Hirano bodies [17-19], though none of these studies focused on Hirano bodies. The physiological effect of Hirano bodies is not understood, since nearly all prior reports on Hirano bodies have been performed on post-mortem samples by immunohistochemistry and electron microscopy.

We have developed cell models for the formation of Hirano bodies in the cellular slime mold *Dictyostelium discoideum *as well as in a variety of cultured cell models such as fibroblasts, epithelial cells, glial cells, neuronal cell lines, and primary neurons by expressing a truncated form (C-terminal amino acids 124-295) of the 34 kDa F-actin bundling protein [14,20-23]. The model Hirano bodies closely mimic authentic Hirano bodies in the brain both in composition, and in ultrastructural characteristics [14,22,23]. Specifically, these model Hirano bodies are paracrystalline filament arrays that contain actin, actin binding proteins, tau, and C-terminal fragment(s) of amyloid precursor protein as previously reported in human and animal tissues.

To understand the physiological function and effect of Hirano bodies in aging and disease progression, we have used the Cre/loxP system to generate a transgenic mouse model expressing CT-GFP flanked by loxP sites at the Rosa26 locus [24-26]. In this study, we have confirmed that CT-GFP was expressed in the brain from day 14.5 following Thy1-Cre recombination, consistent with previous characterization of this promoter [27]. However, the formation of Hirano bodies in the hippocampus was first observed in 6 month homozygous double transgenic mice (R26CT^+/+^;Cre^+^). The presence of these inclusions was confirmed using light and electron microscopy as eosinophilic rod-shaped structures, and as paracrystalline F-actin structures consisting of 8-10 nm filaments with 10-12 nm spacing. This mouse model for Hirano bodies may provide a valuable tool to study the effects of Hirano bodies in human disease and aging.

## Methods

### Mice

**Figure 1:**
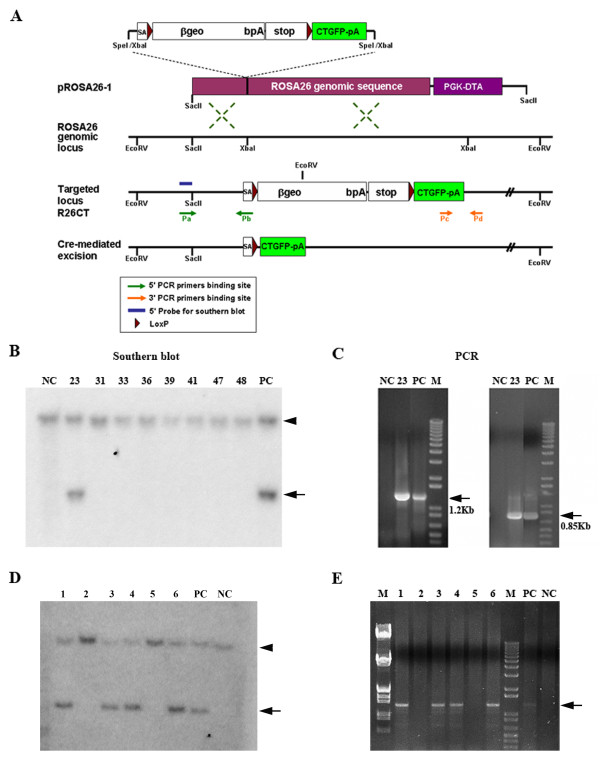
**Targeting strategy of CT-GFP inducible R26CT mouse**. (A). Top: The structure of targeting vector containing a loxP-flanked (bgeo/stop) cassette with CT-GFP pA target gene. A pROSA26-1 plasmid contains ROSA26 genomic sequences and a diphtheria toxin gene (PGK-DTA) to increase rate of homologous recombination and negative selection in ES cells, respectively. Middle: the genomic organization of endogenous Rosa26 locus. The structure of the targeted Rosa26 allele (R26CT) which expresses β-galactosidase and a neomycin resistance cassette fusion gene mRNA. Bottom: Cre-mediated excision of targeted R26CT allele to transcribe CT-GFP mRNA. loxP, solid arrow head; βgeo, a fusiongene of β-galactosidase and neomycin (Neo) resistant gene;. pA, polyA signal; STOP, a transcription stop sequence. (B) Southern Blot analysis of genomic DNA prepared from Neo resistant ES cell lines. It was digested with EcoRV and hybridized with a^32^P-labeled 5' probe indicated in A. The 3.8 kbp EcoRV fragment indicates the targeted R26CT allele and the 11 kbp EcoRV fragment represents wild type Rosa26 allele. (C) PCR screening for the targeting event. A set of primers shown in A were used to amplify 1.2 kbp (Pa and Pb) and 0.85 kbp (Pc and Pd) size fragments from the R26CT allele, but not wild type Rosa26 allele. (D) Southern Blot analysis of genomic DNA prepared from heterozygous R26CT and wild type mice. After EcoRV digestions, the 5' probe was used the same as mentioned in A. (E) PCR screening for the targeting event to amplify 1.2 kbp band from the R26CT allele.

To generate inducible R26CT transgenic mice expressing CT-GFP, a targeting vector was constructed by inserting a SA-loxP-βgeo-pA-STOP-loxP-CT-GFP-pA CT-GFP inducible cassette into the XbaI site of the vector pROSA26-1, which contains a 5-kb genomic fragment of Rosa26 locus for homologous recombination and a diphtheria toxin (DTA) expression cassette for negative selection [25]. The construct CT-GFP inducible cassette was generated from plasmid pSAβgeo [28] by inserting a loxP site into the HindIII site and ligation with STOP [29] and a loxP-CT-GFP-pA fragment. A CT-GFP-pA fragment was generated by PCR in which the CT fragment (amino acids 124-295) encoding 34 kDa actin bundling protein from *Dictyostelium *was subcloned into the BamHI site of the pEGFP-N1 (Clontech, Palo Alto, CA) at the carboxyl-terminus to express a fusion protein of CT with GFP (CT-GFP-pA). The targeting vector was linearized and introduced into a C57BL/6J mouse ES cell line by electroporation at the Medical College of Georgia Transgenic and Knockout Mouse Core Facility at Augusta, Georgia. Correctly targeted cell lines were screened with PCR and Southern Blot with a 5' flanking probe, both of which have been described previously [25]. For transgene genotyping in ES cells, we used the following primers (Figure 1): Pa (ROSA26 forward - 5' flanking) 5'-CCTAAAGAAGAGGCTGTGCTTTGG-3'; Pb (Rosa SA reverse) 5'-CATCAAGGAAACCC TGGACTACTG-3'; Pc (GFP forward) 5'-GCACCATCTTCTTCAAGGACGAC -3'; Pd (ROSA26 reverse - 3' flanking) 5'-CCGACAAAACCGAAAATCTGTG-3'. This genotyping PCR amplifies a 1.2 Kb band with Pa and Pb in 5' junction, as well as a 850 bp band with Pc and Pd in 3' junction to confirm homologous recombination in ROSA 26 locus using DNA isolated from ES cells. The targeted ES cells were microinjected into blastocysts to generate chimeric mice. The R26CT colony was maintained by PCR genotyping, which can distinguish between the endogenous and transgenic Rosa26 allele. R26CT colony was maintained on C57BL/6J genetic background.

To generate double transgenic mice for the formation of Hirano bodies in the brain, R26CT mice were mated to Thy1-Cre mice (line 703; Dr. Valerie Wallace, University of Ottawa) that express CRE recombinase broadly in neural and non-neural tissues under control of a modified Thy1 promoter [27]. For R26CT and Cre genotyping, we used the following primers: P1 (R26-1GTFOR) 5'-TTGGAGGCAGGAAGCACTTG -3'; P2 (ROSASAREV) 5'-CATCAAGGAAACCC TGGACTACTG-3'; and P3 (R26-1GTREV) 5'-CCGACAAAACCGAAAATCTGTG-3'. This genotyping PCR amplifies a 230 bp band from the R26CT allele and a 369 bp band from the wild type Rosa26 allele. To check for Cre, the primers were: Cre forward 5'-CCAGGCCTTTTCTGAGCATACC-3' and Cre reverse 5'-CAACACCATTTTTTCTGACCCG-3'. The PCR product is 641 bp. The day of the vaginal plug was designated as E0.5. All experiments were carried out with the approval of the University of Georgia institutional animal care committee.

### Immunofluorescence labeling

Immunofluorescence microscopy was performed following methods described previously [30]. Mice from postnatal 0 to 9 month were anesthetized and whole brains were dissected for immunostaining and histology. For cryosection, dissected whole brains were fixed with 4% paraformaldehyde overnight, followed by cryoprotection in 30% sucrose and embedding in OCT (Optical Cutting Temperature, Tissue-Tek 4583) and storage in liquid nitrogen. Sections (10 μm) were cut from frozen tissue using a cryostat (Leica) and electrostatically attached to Super-frost glass slides (Fisher Scientific, Pittsburgh, PA). Sections were blocked for 1 hour in 4% milk (NDM)/TST buffer (10 mM Tris-HCl, pH 7.4; 150 mM NaCl; 0.1% Tween20). They were incubated in primary antibody at room temperature overnight. The slides were washed three times in 4% milk/TST buffer for 5 min each, followed by 1 hour incubation with biotinylated secondary goat anti-rabbit antibody (Jackson Immunoresearch Laboratories, West Grove, PA; 1:100 dilution). After washing as above, they were incubated in Cy2 conjugated streptavidin (Jackson Immunoresearch Laboratories, West Grove, PA; 1:200 dilution) for 30 min. Signal was detected by either standard fluorescence microscopy or laser scanning confocal microscopy. The following antibodies and dilution were used: polyclonal rabbit anti-GFP antibody (Invitrogen, Carlsbad, CA; 1:100 dilution) or polyclonal rabbit anti-34 kDa (1:50 dilution) and Phalloidin-TRITC (Sigma-Aldrich Chemical Co., St. Louis, MO; 1:40 dilution).

### Histology

H and E staining was performed as previously described with slight modification [31]. For paraffin sections, dissected brains were fixed with 4% paraformaldehyde at 4°C overnight, dehydrated in a graded series of 50, 75, 90, 96 and 100% ethanol, equilibrated with xylene, embedded in paraffin and sectioned on a sliding microtome at a thickness of 5-10 μm. After dewaxing with xylene, sections were stained with Mayer's hematoxylin (Sigma-Aldrich Chemical Co., St. Louis, MO) and eosin (Sigma-Aldrich Chemical Co., St. Louis, MO) solution and mounted on slides.

### Transmission Electron Microscopy

TEM was performed as previously described with slight modification [17]. Mice from postnatal 0 to 6 months were anesthetized, and whole brains were dissected to separate hippocampus from cortex and thalamus. Hippocampal tissue blocks were fixed by immersion with 4% paraformaldehyde/2% glutaraldehyde in 0.1 M cacodylate buffer, pH 7.4 overnight, and then postfixed in 1% osmium tetroxide (OsO_4_) for 2 hours. After serial dehydration in ethyl alcohol, tissues were embedded in Epon (Embed-812; Electron Microscope science, PA, USA). Semithin sections were stained with 1% toluidin blue in 1% sodium tetraborate. Ultrathin sections were collected on nickel grids, and then counterstained with uranyl acetate for 30 min and lead citrate for 5 min at room temperature. Samples were observed with a JEOL 100CX with an accelerating voltage of 80 kV.

### Western blot analysis

Western analysis was performed as described previously with slight modification [19]. The brains from transgenic mice were dissected, and hippocampi were homogenized in 5 volumes of lysis buffer containing 1% triton-X 100, 0.1% SDS, 0.5% deoxycholic acid, 20 mM Tris-Cl pH 7.5, 10% glycerol, 0.5 M EDTA, 2 mM PMSF (phenylmethyl sulfonyl fluoride), leupeptin (1 μg/ml), pepstatin (1 μg/ml), and aprotinin (1 μg/ml). Cell debris was separated from total homogenate by centrifugation at 14,000 g for 30 min at 4°C. Supernatant was stored at -80°C until used. Protein concentrations of the supernatants were determined by Bradford protein assay (Bio-Rad, Richmond, CA, USA) using BSA as a standard. For immunoblot analysis, tissue samples were loaded with 100 μg of protein per lane and separated on 12% SDS-polyacrylamide gels. Blots were probed using anti-34 kDa (B2C) mouse monoclonal antibody at a 1:5000 dilution, anti-α Tubulin mouse antibody (Sigma-Aldrich Chemical Co., St. Louis, MO) at a 1:1000 dilution, and anti-MAP LC3 (N-20) goat antibody (Santa Cruz Biotechnology, Inc., Santa Cruz, CA) at a 1:500 dilution. The signals were detected by chemiluminescence (Pierce Biotechnology, Rockford, IL).

### Extracellular Electrophysiology and Quantification of synaptic plasticity

Hippocampal slices were prepared from male homozygous transgenic mice at 7 months of age (R26CT^+/+ ^as control and R26CT^+/+^;Cre^+ ^as Hirano Body animals) using an experimental protocol performed in compliance with the University of Georgia Animal Care and Use guidelines. All mice were anesthetized with halothane prior to decapitation. The brain was removed and submerged in ice-cold, oxygenated (95% O_2_/5% CO_2_) dissection artificial cerebrospinal fluid (ACSF) containing 120 mM NaCl, 3 mM KCl, 4 mM MgCl_2_, 1 mM NaH_2_PO_4_, 26 mM NaHCO_3_, and 10 mM glucose. Horizontal brain slices were cut at a thickness of 400 μm, and the hippocampus dissected free. Slices were perfused with room-temperature, oxygenated (95% O_2_/5% CO_2_) standard ACSF containing 120 mM NaCl, 3 mM KCl, 1.5 mM MgCl_2_, 1 mM NaH_2_PO_4_, 2.5 mM CaCl_2_, 26 mM NaHCO_3_, and 10 mM glucose at approximately 1 ml/min. Slices recovered for one hour at room temperature, and another hour at 30°C, the temperature at which recordings were obtained. A bipolar stimulating electrode (Kopf Instruments, Tujunga, CA) was placed on the CA3 side of the stratum radiatum and an extracellular recording microelectrode (1.0 MΩ tungsten recording microelectrode, (World Precision Instruments, Sarasota, FL) was positioned in the same layer in CA1.

Data were digitized at 10 kHz, low-pass filtered at 1 kHz, and analyzed with pCLAMP 9.2 software (Axon Instruments, Sunnyvale, CA). The initial slope of the population fEPSP was measured by fitting a straight line to a 1 msec window immediately following the fiber volley. Stimulus-response curves were obtained at the beginning of each experiment, with stimulus pulses consisting of a single square wave of 270 μs duration delivered at 30, 40, 50, 60, 70, 85, 100, 120, 140, and 160 μA in the stratum radiatum once every 60 s (0.0167 Hz). To begin baseline recording, the stimulation intensity was adjusted to obtain a field EPSP of approximately 40-50% of the maximum response, and paired-pulse responses were measured from an average of five pairs of pulses delivered at a 50 msec interval. For long-term potentiation (LTP) experiments, synaptic responses were normalized by dividing all fEPSP slopes by the average of the 5 responses obtained from the 5 min immediately prior to high-frequency stimulation (HFS). The HFS protocol used to induce LTP in all experiments consisted of 3 episodes of 100 Hz/1 sec stimulus trains administered at 20 sec intertrain intervals. Planned comparisons between homozygous R26CT^+/+ ^transgenic mice as controls were made with R26CT^+/+^;Cre^+ ^Hirano Body mice at 30 minutes and 240 minutes post-HFS using unpaired t-tests. Reported n-values (x(y)) indicate the number of slices (x) and the number of animals (y) assessed.

## Results

### Generation of R26CT transgenic mice

To generate a transgenic (Tg) mouse model of Hirano bodies, we anticipated possible lethality and so devised a strategy to target the broadly expressed and weak ROSA promoter in embryonic stem cells, and employed CRE technology to allow flexibility in control of tissue specific expression. We prepared a targeting vector, in which the sequence encoding CT-GFP along with poly A was inserted downstream of a floxed βgeo-pA-STOP cassette, and introduced into the pROSA26-1 trap vector. The pROSA26-1 trap vector contains ROSA26 genomic sequences to permit homologous recombination and also a PGK-DTA cassette (phosphoglycerate kinase-1 promoter and diphtheria toxin) for negative selection (Figure 1A) [25]. The targeting vector was electroporated into embryonic stem (ES) cells derived from strain C57BL/6 and the targeted alleles were screened by Southern blotting to determine homologous recombination (Figure 1A, B). To confirm homologous recombination of CT-GFP transgene within the ROSA26 locus, 5' and 3' flanking regions were amplified by PCR using two primer sets at the same time (Figure 1A, C). The targeted ES clones were injected into C57BL/6 blastocysts to generate chimeric mice. By testing these chimeric animals for germ line transmission, we identified 4 founders (R26CT) out of 6 mice in which the CT-GFP transgene was targeted into ROSA26 genomic regions as determined by southern blot and PCR (Figure 1D, E).

### Generation of R26CT^+/-^;Thy1-Cre^+ ^double transgenic (DTg) mice

To induce formation of Hirano bodies, we used the Cre/loxP site specific recombination system [25,26] in which the tissue specificity of expression for the recombination-activated dormant transgene is determined by the promoter specificity of the Cre transgene. Thy1-Cre transgenic mice induce a transgene with tissue-specificity in the central and peripheral nerve systems including neuronal tissues of the cerebral cortex and hippocampus, and non-neuronal tissue [27]. R26CT transgenic mice carrying CT-GFP transgene were mated with Thy1-Cre (703 line) transgenic mice expressing the Cre recombinase under the control of murine Thy1.2 regulatory elements. Homozygous R26CT (R26CT**^+/+^; **Cre^+^) double transgenic mice were generated through appropriate crosses between heterozygous R26CT (R26CT**^+/-^; **Cre^+^) mice to increase the expression level of the CT-GFP transgene. Transgene and Cre gene presence as well as copy number were determined by PCR genotyping as described in Materials and Methods.

### Expression of CT-GFP in the hippocampus at P0

**Figure 2:**
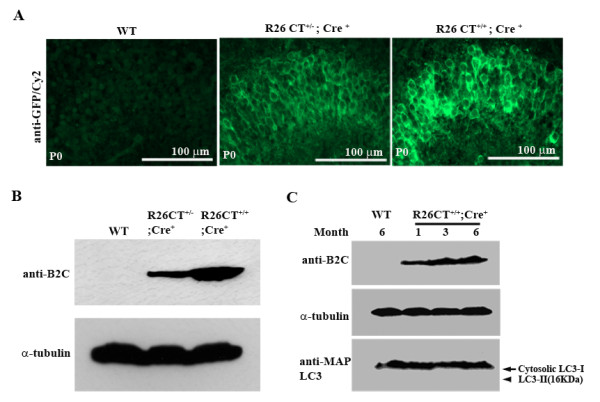
**Expression level of transgene CT-GFP in the brain of double transgenic R26CT allele**. (A) Transgene expression in the hippocampus of double transgenic R26CT mouse and wild type (WT) at P0. Heterozygous and homozygous double transgenic mice were stained with anti-GFP antibody. (B,C) Homogenates of hippocampi of different double transgenic R26CT allele were analyzed by Western blot. Expression of CT-GFP was correlated with transgene copy number, increased from 1 to 6 month of age. Major induction of autophagy as indicated by accumulation of the modified form of LC3-I was not detected. Arrow indicates cytosolic LC3-I and arrow head represents an autophagosome-associating form, LC3-II which is an autophagy marker.

Since Thy1-Cre reporter activity was observed in retina and hippocampus at postnatal day 0 (P0) in Thy1-Cre;ROSA transgenic mice [27], we asked whether double transgenic mice express CT-GFP in the hippocampus at P0. To assess transgene expression in hippocampus of the brain, frozen sections of the brain prepared from neonatal wild type, heterozygous, and homozygous transgenic mice were stained with antibody to GFP to enhance fluorescence from the transgene, and visualized by fluorescence microscopy. CT-GFP transgene was expressed in the hippocampus (Figure 2A), consistent with prior characterization of the Thy1-Cre model [27]. The expression level of CT-GFP of homozygous DTg mice was higher than that of heterozygous DTg mice (Figure 2A). To confirm these results using a biochemical approach, lysates of hippocampi of each genotype of DTg mice were examined by western blot analysis with anti-34 kDa (B2C) antibody (Figure 2B). Expression levels of the transgene in homozygous DTg mice were increased more than two times compared to that of heterozygous DTg mice. Therefore, the expression level of CT-GFP protein is consistent with transgene copy number. Further, from 1 to 6 months of age, the level of expression of the CT-GFP protein increased (Figure 2C).

**Figure 3:**
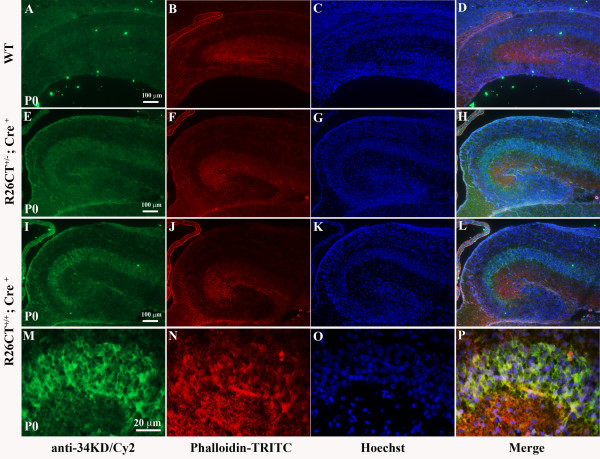
**Expression of transgene CT-GFP in the hippocampus**. Sections cut through brain of wild type (A-D) and R26CT allele (E-P) were visualized by immunofluorescence at postnatal 0 (P0). Hirano bodies induced by CT-GFP protein are co-localized with phalloidin in the pyramidal layer of hippocampus. Brain sections are stained with anti-34 kDa (green; A, E, I, M) to detect CT-GFP protein or with phalloidin TRITC-conjugated (red; B, F, J, N) to detect F-actin, Hoechst 33342 (C, G, K, O) for nuclei, and merged images (D, H, L, P). (A-D) wild type. (E-H) heterozygous R26CT^+/-^;Cre^+^. (I-P) homozygous R26CT^+/+^;Cre^+^. (M-P) Higher magnification of hippocampal cells in homozygous double transgenic mice. Areas of co-localization appear yellow in the merged image. Scale bar = 100 μm (A-L), 20 μm (M-P).

It has been reported that the major component of Hirano bodies is F-actin [5]. To examine whether CT-GFP co-localizes with F-actin in the brain at P0, expression of CT-GFP in the hippocampus was examined by immunohistochemistry using either anti-34 kDa antibody (Figure 3) or anti-GFP antibody (data not shown) in combination with phalloidin to stain filamentous actin. We confirmed that CT-GFP was not only predominantly expressed in the hippocampus, but also largely co-localized with F-actin in the hippocampal pyramidal layer CA1, CA2, and CA3 regions. In addition, the expression level of CT-GFP was higher in CA3 as compared to CA1 (Figure 3).

### Formation of Hirano bodies in the hippocampus of 6 month homozygous DTg mice

In various human disorders and experimental animal models, Hirano bodies have two major distinctive features: first, eosinophilic rod-shaped cytoplasmic inclusions that contain actin filaments [1,5]; and second, lattice-like aggregates of parallel filaments [2,3]. These features were utilized to test for formation of Hirano bodies in our transgenic mice.

**Figure 4:**
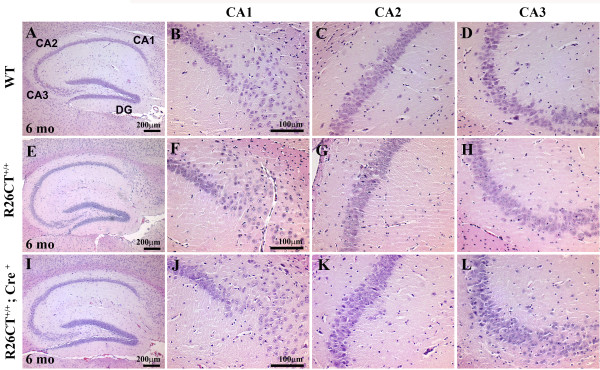
**Histological analysis of 6 month adult brains**. Serial sagital sections of brains from wild type and homozygous transgenic mice were analyzed by hematoxylin and eosin (H and E) staining. (A-L) No differences in the pyramidal cell layer in the hippocampus (CA1, CA2, and CA3) are discernable between wild type (A-D), R26CT^+/+ ^(E-H), and R26CT^+/+^;Cre^+ ^(I-L) transgenic mice at 6 months of age.

**Figure 5:**
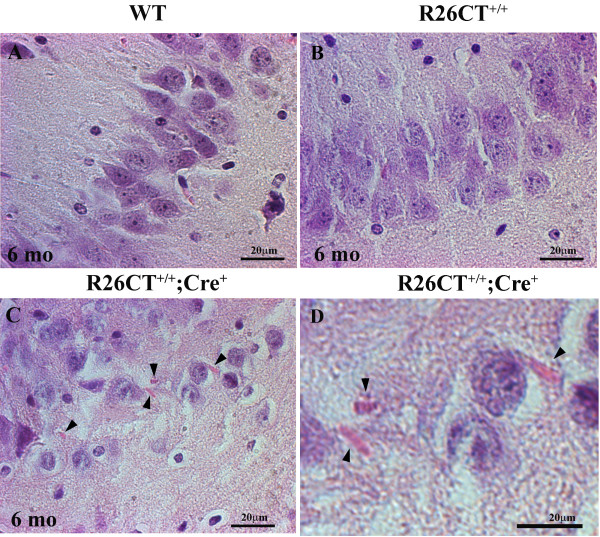
**Detection of Hirano Bodies by H and E Staining**. The rod-shaped eosinophilic inclusions were found in the vicinity of pyramidal cells of hippocampus in homozygous double transgenic mice (C,D). Hirano bodies were not observed in wild type mice (A), or double transgenic mice lacking Cre (B). Arrow heads indicate Hirano bodies. Scale bar = 20 μm.

**Figure 6:**
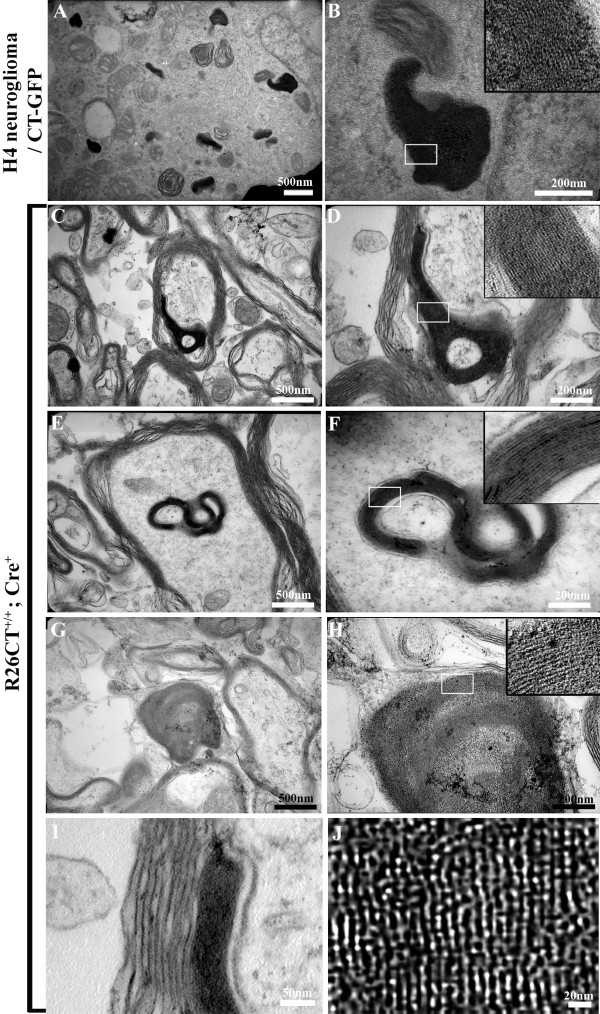
**Paracrystalline structure of mouse model Hirano Bodies**. Hemisphere brain was dissected by separating hippocampus from cortex and thalamus. (A, B) Transmission electron microscope (TEM) images of the electron dense region in CT-GFP stable H4 human neuroglioma cells show the composition, filamentous organization, and cross-hatched appearance of the inclusions. (C-J) TEM images of hippocampus in homozygous double transgenic mice at 6 months reveal model Hirano bodies with characteristic structure and periodicity. The small size of filamentous inclusions are enclosed by myelin sheath and resemble the ultrastructural definition of Hirano bodies reported in the hippocampus of an Alzheimer's patient. Scale bars: 500 nm (A, C, E, G), 200 nm (B, D, F, H), 50 nm (I), and 20 nm (J). White square box shows a filamentous array that is magnified in the inset (B, D, F, and H).

To determine whether model Hirano bodies are present as indicated by the presence of eosinophic rod-like aggregates and paracrystalline structure, we dissected whole brains and performed H and E staining for histology (Figure 4 and 5), and electron microscopy (Figure 6) on the two hemispheres of brains from heterozygous and homozygous transgenic mice. We did not detect the formation of Hirano bodies in any heterozygous (R26CT^+/-^; Cre^+^) transgenic mice (3, 6, 9 and 12 months of age; n = 2 for each age), and in homozygous (R26CT^+/+^; Cre^+^) transgenic mice (1 and 3 months of age; n = 2 for each age; data not shown). By contrast, in homozygous DTg mice 6 months of age, we observed eosinophilic cytoplasmic inclusions adjacent to the perikaryon of pyramidal cells in the hippocampus (Figure 5C, D). Eosinophilic Hirano bodies were not observed in wild type controls even as old as 24 months (data not shown). Further, ultrastructural study showed that Hirano bodies, under 1 μm in diameter, appeared to lie partially within myelinated sheath. These paracrystalline inclusions were composed of parallel filaments 8-10 nm in diameter with 10-12 nm interspacing between filaments consistent with previous studies (Figure 6) [2,32,33]. As a control, we used H4 stable cells expressing CT-GFP [14,22]. Hirano bodies were clearly seen in the cytoplasm without enclosed membrane (Figure 6A and 6B), consistent with prior reports of model Hirano bodies in cultured cells. Thus, Hirano bodies were observed both by H and E and by electron microscopy in homozygous transgenic mice 6 months of age (n = 2 for both H and E and electron microscopy), but not in heterozygous transgenic mice 3, 6, 9, and 12 months of age. Further, the Hirano bodies were observed both by electron microscopy and by H and E staining only in animals with Cre recombinase, and never in Cre^- ^animals (Figure 5 and 6; data not shown).

The absence of Hirano bodies in heterozygous transgenic mice with a single copy of the transgene, and delay in appearance of Hirano bodies until 6 months in homozygous transgenic mice in vivo was surprising to us, since formation of Hirano bodies was observed with 24 hours of induction of expression in cultured cell models of Hirano bodies [14,20-22]. Formation of Hirano bodies in homozygous double transgenic mice but not in heterozygous double transgenic mice with only a single copy of the transgene may be due to the low level of expression in mice carrying a single copy of the transgene (Figure 2B). This finding is consistent with the observation that the Rosa26 promoter has been characterized to be a ubiquitous promoter which is relatively weak and displays a low level of transcription [34].

To understand why the formation of Hirano bodies is delayed to 6 months in the homozygous double transgenic mice with two copies of the transgene, we performed western blot analysis using hippocampi dissected from homozygous DTg mice 1, 3, and 6 months of age to determine their levels of expression of the 34 kDa protein. Between 1 and 6 months, expression of CT-GFP protein increased (Figure 2C), suggesting that the accumulation of the CT-GFP protein could contribute to modulation of the formation of Hirano bodies. Since autophagy can contribute to the degradation of Hirano bodies in cultured cells [23], we tested for induction of autophagy in our Hirano body model mice. We used Western blotting to examine the accumulation of two forms of the autophagy protein termed LC3-1 and LC3-II (Figure 2C). Increased autophagic activity is reflected by the enhanced conversion of LC3-I to LC3-II [35]. Expression of LC3-II was not detected by 6 month (Figure 2C, arrow head). However, we found consistent expression of cytosolic LC3-I protein in the brain (Figure 2C, arrow). While these findings do not rule out the role of basal autophagy in control of the levels of Hirano bodies in the brain, they do show that no major induction of autophagy is detectable in these mice.

### Effects of Hirano body expression on hippocampal structure and physiology

Both heterozygous and homozygous DTg mice were viable and fertile (data not shown). To determine whether the presence of Hirano bodies affected the number and organization of hippocampal pyramidal neurons, we compared preparations from wild type and double transgenic mice by H and E staining. There was no difference in cell density or organization in the CA1, CA2, or CA3 regions of the hippocampus among WT, R26CT^+/+^; Cre^+^, and R26CT^+/+^; Cre^- ^mice (Figure 4A-L).

**Figure 7:**
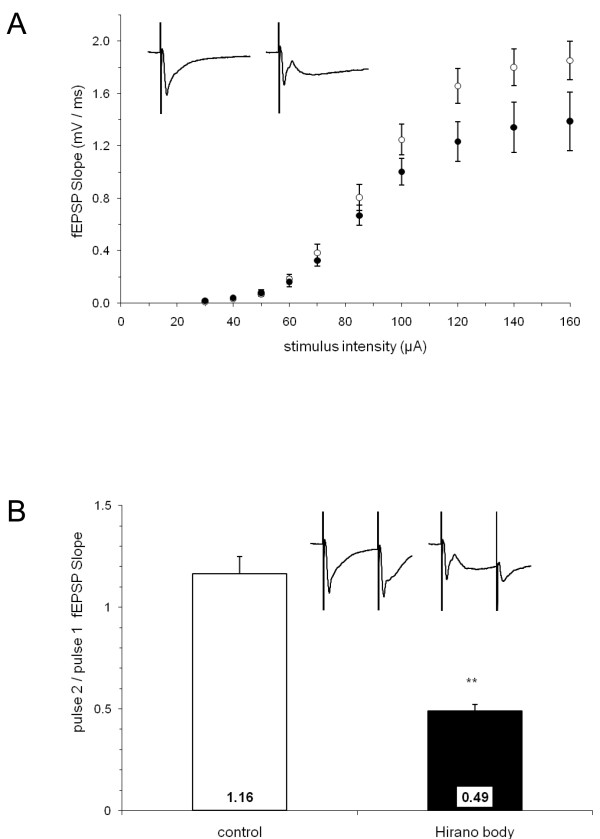
**Synaptic fEPSP responses evoked and recorded in the s. radiatum layer of Hirano Body and control mice**. Insets are averaged fEPSP sweeps for both control and Hirano Body groups. The stimulus artifacts are truncated at 3 mV in the vertical axis, and the sweeps are 100 msec in duration in the horizontal axis. A) Stimulus-response curves for both Hirano Body (filled circles) and control mice (open circles) at the indicated stimulus intensities. B) Paired-pulse fEPSP responses (50 msec isi). The open bar represents the control group (n = 13(7)); the filled bar represents the Hirano body group (n = 8(4)). The paired-pulse ratios were determined by dividing the slope of the second pulse by that of the first (**, p < 0.001, unpaired t-test). Error bars show mean ± SEM.

In order to determine if the presence of Hirano Bodies can impact neuronal function at 7 months of age, field excitatory post-synaptic potentials (fEPSPs) were recorded in the stratum radiatum layer as a measure of synaptic function in the CA1 region of the hippocampal formation. Although a tendency for the baseline fEPSPs of Hirano body mice to be smaller than those recorded from control mice was evident, the responses of these groups were not significantly different at any of the tested stimulus intensities (Figure 7A). We also investigated the effect of a paired-pulse stimulus protocol in which a second stimulus pulse (P2) was delivered 50 msec after the first pulse (P1, Figure 7B). Control R26CT^+/+ ^mice exhibited paired-pulse facilitation (P2/P1 ratio of 1.16 ± 0.09; n = 13(7)), consistent with the expected responses from wild type mice [36]. Unexpectedly, a strong paired-pulse depression (P2/P1 ratio of 0.49 ± 0.03; n = 8(4)) was observed in the Hirano body mice (**, p < 0.001, unpaired t-test).

**Figure 8:**
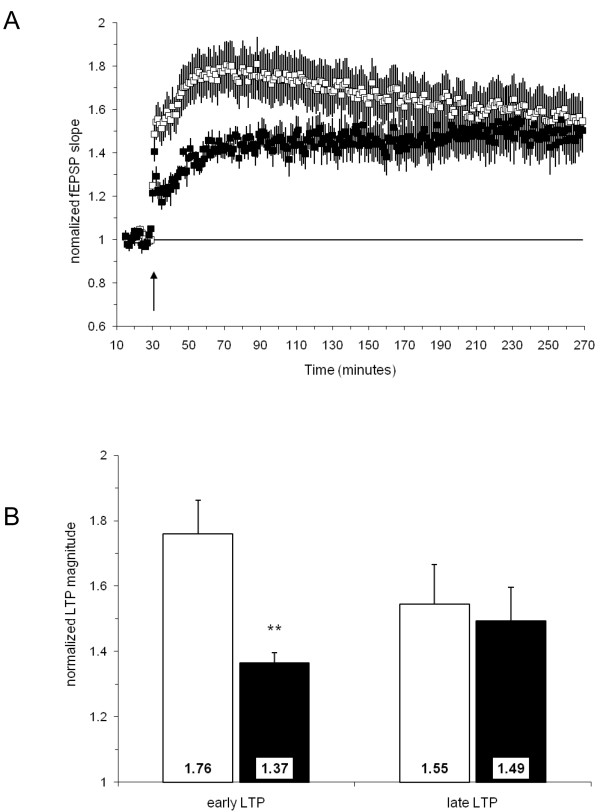
**Long-term Potentiation of the fEPSP responses from Hirano Body and Control Mice**. A) Summary plot of normalized fEPSP slope measurements evoked and recorded in the s. radiatum layer of the CA1 region. Open squares depict responses from the control group; closed squares represent responses from the Hirano body group; HFS (3 × 100 Hz/1 sec) was administered at time = 30 min (arrow) to induce LTP. B) Summary quantification of LTP magnitude at both 30 min post-HFS (early LTP) and 240 min post-HFS (late LTP). The open bars represent the control group; the filled bars represent the Hirano body group (**, *p *< 0.01, unpaired t-test). Error bars show mean ± SEM.

Following the induction of long term potentiation (LTP), the fEPSP slope at 30 minutes post-tetanus was increased 76 ± 10% in the hippocampal slices of control mice (homozygous R26CT^+/+^; Cre^-^; n = 13(7)) as compared to 37 ± 3% in transgenic mice (homozygous R26CT^+/+^; Cre^+^; n = 8(4)), and this difference was statistically significant (**, p < 0.01, unpaired t-test). However, at 240 min post-tetanus the fEPSP slope was not significantly different in transgenic Hirano body mice compared to those of control mice (55 ± 12%, 49 ± 10% respectively, Figure 8A,B).

Taken together, the electrophysiology results suggest that Hirano bodies are not toxic, that baseline synaptic function in the CA1 region of the hippocampus is preserved in Hirano body mice, and that these synapses can exhibit activity-dependent plasticity in the form of late LTP measured up to four hours post-tetanus. However, there are obvious differences in more transient forms of synaptic plasticity from those observed in the control mice. Notably, there is a shift from facilitation to depression in the paired-pulse assay during short-term plasticity, and a reduction in the magnitude of early LTP assessed 30 minutes post-tetanus during induction of long-term plasticity. Studies of homozygous double transgenic (R26CT^+/+^; Cre^+^) and control (R26CT^+/+^; Cre^-^) mice at 18 months of age produced results comparable to those observed at 7 months, but the short supply of these aged mice severely limited these observations (data not shown).

## Discussion

Our knowledge of Hirano bodies includes a careful description of their structure, histology, and association with a variety of conditions including normal aging and neurodegenerative disease [4]. However, we have very little information regarding the effect(s) of these structures on aging and disease progression in living cells. In this report, a mouse model of Hirano bodies was generated in order to permit investigation of Hirano bodies within a multicellular vertebrate organism. The authenticity of the Hirano bodies was verified by comparison to the hallmark properties reported consistently in prior studies of Hirano bodies. Eosinophilic rod-shaped inclusions were seen in the CA3 region of the hippocampal pyramidal cell layer by H and E staining (Figure 5) [1]. The CT-GFP was largely co-localized with rhodamine-phalloidin confirming the presence of a high concentration of actin filaments (Figure 3) [5]. These structures were detected only in mice that contain both the CT-GFP transgene and the CRE recombinase, and were also enriched for the CT-GFP transgene that we used to induce the model Hirano bodies (Figure 3). Finally, images from electron microscopy confirmed the presence of paracrystalline structures with a filament diameter of 8-10 nm and a center to center spacing of 10-12 nm that were surrounded by a myelin sheath. These inclusions closely resemble the ultrastructural features of Hirano bodies reported in cultured cells and in the human hippocampus (Figure 6) [2,32,33]. Further, the presence of Hirano bodies within myelinated fibers is also consistent with prior reports [4,32]. These findings demonstrate that the model Hirano bodies in this transgenic mouse closely mimic the features of Hirano bodies reported in humans and animal models.

While our results clearly demonstrate the presence of model Hirano bodies in the hippocampus of the mice, a number of interesting questions regarding the formation of these structures remain to be addressed. First, the formation of Hirano bodies was initially observed in homozygous double transgenic mice (R26CT^+/+^;Cre^+^) aged 6 months, but not in younger mice of the same genotype nor in mice carrying a single copy of the transgene (R26CT^+/-^; Cre^+^). Although we were able to detect CT-GFP expression by immunohistochemistry in the brain of these heterozygous mice (R26CT^+/-^; Cre^+^) from P0 to 12 months of age (the oldest age studied), the formation of Hirano bodies was not detected. These findings were surprising, since we have consistently observed the formation of Hirano bodies in cell cultures within one day of expression of the transgene [14,20-22].

One explanation for these results is that the level of CT-GFP expressed in this mouse model is extremely low. The ubiquitous ROSA26 promoter is relatively weak and expresses a very low level of transcription of transgene [34]. Further, a single copy of transgene may not be enough to induce the formation of Hirano bodies at this low level of transcription. The increase in level of CT-GFP between 1 and 6 months of age may promote the formation of Hirano bodies (Figure 2C). More generally, the frequency of Hirano bodies found in the neurons of normal individuals as well as subjects with a number of neurodegenerative diseases including Alzheimer's disease is known to increase with age [33]. Thus, although Hirano bodies form within 24 hours after expression in cultured cells [14,22], other factors may contribute to the delay of 6 months in formation of Hirano bodies in the brain. Since autophagy and the proteasome both contribute to the turnover of Hirano bodies [23], it seems possible that these degradative mechanisms may contribute to this delay. However, our findings did not support the hypothesis that autophagy is induced in these animals and delays the accumulation of Hirano bodies (Figure 2). Efficient degradation of CT-GFP by the proteasome [23] or basal levels of autophagy may be responsible for the significant lag between expression of the CT-GFP protein and the appearance of Hirano bodies.

The precise mechanisms that induce formation of Hirano bodies in the brain remain unknown. In prior published reports, Hirano bodies are described as large well ordered structures 10-20 μm in diameter [5,32]. In cultured cells, formation of model Hirano bodies occurs by aggregation of smaller structures into larger ordered aggregates [22,37]. The accumulation of smaller ordered structures into a single aggregate initially gives rise to a fingerprint appearance in our culture model [22], which corresponds in appearance to fingerprint inclusions described previously [38]. We also observed smaller Hirano bodies (~1 μm) in this mouse model (Figure 6). These findings suggest that Hirano bodies are formed by a process in which small ordered aggregates form, coalesce into larger structures, and then rearrange into a single large ordered array of the type most often reported in the literature. Recent reports implicate tau [39], altered forms of VASP [40], and stabilization of actin filaments [41-43] as factors in the formation of Hirano bodies. However, the structures formed in these studies lack the paracrystalline organization of authentic Hirano bodies. Identification of the critical trigger or inducer of formation of Hirano bodies in vivo is a significant challenge for future studies.

While Hirano bodies are known to be associated with a large number of conditions and diseases including aging, chronic alcoholism, and neurodegenerative disease, we know very little regarding the potential effects of Hirano bodies in promoting and/or modulating the progression of disease. Actin is a major cytoskeletal protein in neurons that is involved in many aspects of cell motility, vesicle transport, and synaptic plasticity. Rearrangement of the cytoskeleton has been implicated in cellular response to stress and development of pathology in neurodegenerative disease [44]. Our prior studies show that cultured cells with Hirano bodies grow at rates comparable to or slightly slower than those of control cells [20-22]. Further, Hirano bodies modulate transcription dependent on AICD and Fe65 and attenuate AICD induced cell death in cell cultures [14], suggesting that Hirano bodies may function as endogenous protective structures that delay or prevent development of pathology. The results in the present work reveal that the presence of Hirano bodies in pyramidal cells in the hippocampus does not grossly affect the organization or number of these cells (Figure 4). Homozygous double transgenic mice develop Hirano bodies, but remain healthy and fertile with no apparent behavioral phenotypes. At the functional level, basal synaptic transmission following a single stimulus was not significantly altered (Figure 7A), although short-term synaptic plasticity in response to paired-pulse stimulation was dramatically shifted from facilitation to depression (Figure 7B). In addition, late LTP was observed in hippocampal slices from mice with model Hirano bodies of a magnitude that did not differ significantly from that of wild type mice, whereas the magnitude of early LTP was significantly decreased (Figure 8).

There are a number of possible explanations for these effects of Hirano bodies on synaptic plasticity. First, the sequestration of actin in Hirano bodies may affect the diverse roles of the actin cytoskeleton in multiple pre- and postsynaptic processes [45]. Hirano bodies sequester a large amount of actin that is assembled in filaments, resulting in an increase in the level of filamentous actin, a decrease in unpolymerized actin, and no change in total cellular actin [20,21]. The facilitation of synaptic response observed following paired-pulse stimulation is thought to reflect a presynaptic event related to residual calcium at the synapse [46]. Following the first stimulus event, recovery involves not only active transport of calcium, but also trafficking of neurotransmitter-containing vesicles in order to regenerate a pool of neurotransmitter containing vesicles that is competent for rapid exocytosis and release following elevation of calcium [47]. Since the trafficking of these neurotransmitter containing vesicles is also modulated by the actin cytoskeleton [48], the simplest explanation for the observed paired-pulse depression is a change in vesiclular trafficking due to the presence of the Hirano body. Another report of paired-pulse depression in mice that are deficient for profilin, a potent facilitator of actin assembly, is consistent with this interpretation [49].

Long-term potentiation has two phases, early and late, both of which are associated with an increase in actin assembly and only the latter requires protein synthesis [50-52]. The actin assembly associated with the late stage of LTP involves an increase in the number and size of dendritic spines, and growth of the postsynaptic densities [50,51]. The fact that LTP in Hirano body mice reaches the magnitude observed in wild type mice during the last phase (i.e. 3+ hr post induction) demonstrates that the necessary changes in protein synthesis, receptor trafficking and distribution, and spine morphology have all occurred to support the maintenance of late LTP. The actin assembly occurring during the early stage of LTP likely reflects the role of the cytoskeleton in trafficking of NMDA and AMPA receptors [53-55]. Thus, one likely explanation for the deficiency in early LTP observed in the R26CT^+/+^;Cre^+ ^mice is that the available dynamic actin pool is smaller in neurons with Hirano bodies, resulting in a delay in the rate of receptor trafficking and in development of the early phase of LTP. In addition, the alterations in synaptic plasticity in our transgenic mice may be a consequence of the association with Hirano bodies of synaptic components and/or modulators including PSD 95 [56] and atypical protein kinase C isoforms [57]. These potential molecular explanations of the effects of Hirano bodies on synaptic plasticity merit additional study.

Future studies of these mice will be required to determine the behavioral correlates of these changes in short-term and long-term synaptic plasticity in the hippocampus. The depression of paired-pulse responses could have a variety of behavioral/physiological results. One such possibility would be suppression of excitotoxicity in animals with Hirano bodies, as excessive glutamatergic excitation is proposed to contribute to cell death and neurodegeneration [58]. With respect to the effects on long-term plasticity, since LTP reaches normal levels at the later phase, we predict that some tests in Hirano body mice will indicate normal capacity for learning and memory, although deficits related to the impairment of the early phase of LTP may be significant. The behavioral consequences of these diverse results for these various aspects of synaptic plasticity remain to be elucidated. Further, studies of neuropathology, electrophysiology, and behavior at multiple time points will allow correlation of results from these indicators during aging and disease progression.

The mouse model for Hirano bodies described here will be a critical tool for future studies of the role of Hirano bodies in disease progression. Although some APP transgenic mouse strains revealed Hirano-like bodies in the brain, the physiological roles of the Hirano bodies on AD progression could not be determined [17-19]. The ability to generate mice that model both Alzheimer's pathology and model Hirano bodies should provide an avenue to determine directly whether Hirano bodies have either a deleterious or adaptive effect on Alzheimer's disease progression. Similarly, crossing model Hirano body mice with mouse models for other diseases will permit study of their role in the progression of the many different conditions with which they are associated.

## Conclusions

We have developed the first transgenic animal model for experimental studies of Hirano bodies in vivo. The presence of Hirano bodies in the hippocampus was confirmed by H and E staining and by electron microscopy. Transgenic mice with Hirano bodies appear healthy and fertile. No abnormalities in the organization and histology of the brain were detected. Mice with Hirano bodies exhibit distinct alterations in synaptic plasticity. This mouse model will be valuable for future studies of the role of Hirano bodies in aging and in progression of disease.

## List of Abbreviations

LTP: long term potentiation; H and E: hematoxylin and eosin; CT: carboxy-terminus of the 34 kDa actin binding protein; GFP: green fluorescent protein

## Authors' contributions

SH prepared targeting constructs, screened ES cells and mice, managed the mouse colony, characterized the mice by molecular genetics, immunohistochemistry, and electron microscopy, and wrote the initial draft of the manuscript; MS performed the studies of synaptic plasticity on hippocampal slices;

RF helped to train and supervise SH with molecular biology and immunohistochemistry, and in interpretation of data and preparation of figures; JW trained and supervised MS, and participated in analysis of data and preparation of figures for the electrophysiology; MF conceived the project, participated in analysis of data and writing of the draft. All authors contributed to generation of the final form of the manuscript and approved the submission.
